# Photothermal activity in cancer therapy and antimicrobial properties of green gold nanoparticles from winery waste

**DOI:** 10.1039/d6ra01505k

**Published:** 2026-04-17

**Authors:** Lucia Mergola, André F. Moreira, Sónia P. Miguel, Paula Coutinho, Christian Demitri, Antonio Serra, Daniela Manno, Alessandro Buccolieri, Roberta Del Sole

**Affiliations:** a Department of Engineering for Innovation, University of Salento Via per Monteroni 73100 Lecce Italy lucia.mergola@unisalento.it roberta.delsole@unisalento.it; b BRIDGES-Biotechnology Research, Innovation and Design for Health Products, Polytechnic University of Guarda Avenida Dr Francisco Sá Carneiro, No. 50 6300-559 Guarda Portugal; c RISE-HEALTH-Department of Medical Sciences, Faculty of Health Sciences, University of Beira Interior Av. Infante D. Henrique 6200-506 Covilhã Portugal; d Department of Experimental Medicine, University of Salento Via per Monteroni 73100 Lecce Italy; e Department of Mathematics and Physics “E. De Giorgi”, University of Salento 73100 Lecce Italy

## Abstract

Nanomaterial-mediated photothermal therapy is an emerging approach to cancer care; however, the required biocompatibility of nanoparticles remains a critical aspect. To enhance their biocompatibility, biocompatible sources can be used in nanoparticle preparation instead of the conventional toxic substances that are generally required. To this end, grape marc, obtained as agricultural waste from the winery industry, was chosen as an important source of natural compounds. By modifying the nanoparticle surface, it can form a crucial interface with the surrounding environment. In this work, green gold nanoparticles were synthesized and applied in photothermal therapy, and their biocompatibility, antitumor, and antibacterial properties were evaluated. Reducing sugars and polyphenolic compounds, naturally occurring in grape marc waste, were extracted in water and successfully employed in the preparation of gold nanoparticles, as confirmed by the appearance of a plasmonic band at 535 nm. A second peak at 286 nm, typical of phenolic groups, demonstrated the presence of a polyphenolic layer on the nanoparticle surface, which was also confirmed in FTIR studies. TEM and XRD analyses revealed their crystalline nature, with an average diameter of about 30 nm. A third absorption peak in the near-infrared region (750 nm) shows that the synthesized gold nanoparticles can be good photothermal agents with a temperature increase of about 22 °C after 10 minutes of NIR laser irradiation (808 nm and 1.7 W cm^−2^). Good biocompatibility with human fibroblasts and breast cancer cells (MCF-7) was also demonstrated at high concentrations (400 µg mL^−1^), with the cell viability remaining above 70% during 72 hours of incubation. Antibacterial and antitumoral effects of the synthesized nanoparticles were observed after NIR laser irradiation, with 41.7% and 52.5% cell viability inhibition in *S. aureus* and *E. coli*, respectively, at higher concentrations, and a reduction of MCF-7 cell viability to 37%.

## Abstract 2

Green gold nanoparticles, obtained from winery waste, showed good photothermal activity after NIR laser irradiation and their application in the reduction of breast cancer cells and bacteria viability was successfully demonstrated.

## Graphical abstract

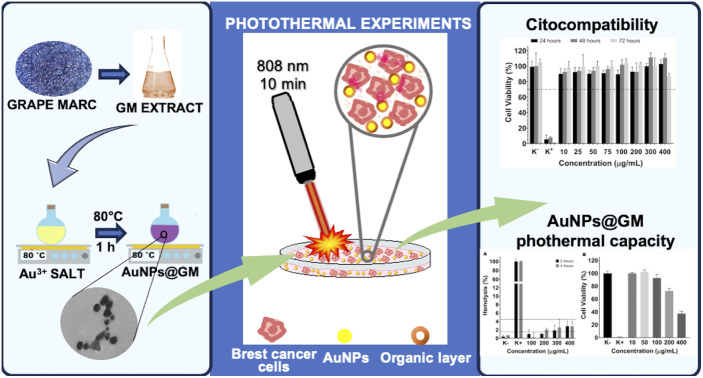

## Introduction

Nanotechnology today represents a new field of science that aims to manipulate matter at the nanoscale level to develop new advanced nanomaterials with unique physical and chemical properties compared to their bulk counterparts. Such materials can be adopted in different fields ranging from engineering to medicine. Nanomedicines provide some of the most interesting and promising applications of nanotechnology by exploiting the peculiar characteristics of nanomaterials for the development of new and more effective therapies, from antibacterial to antitumoral treatments.^1,2^ Nanoparticle-mediated photothermal therapy (PTT) is an emerging treatment that exploits the unique optical characteristics of nanoparticles to generate heat when irradiated with light, such as near-infrared radiation (NIR).^3^ This phenomenon is related to the synchronization of free conductive band electrons that collectively move in resonance with the incident light, causing a strong local electromagnetic field with consequent localized heating.^4^ The confinement of this effect to the irradiated region containing nanoparticles has been harnessed to mediate the elimination of cancerous cells while preserving healthy tissues. For this purpose, the light must be able to penetrate human tissues with minimal interaction. In this regard, three major biological windows can be used to minimize the non-specific damage to the tissues: NIR-I (700–950 nm), NIR-II (1000–1350 nm), and NIR-III (1550–1870 nm).^5^ The choice of the light source depends on the specific photothermal agent used.^6^ A lot of nanomaterials, such as carbon dots,^7^ metal and metal oxide nanoparticles,^8^ noble-metal nanoparticles^8,9^ and carbon nanotubes,^10^ have been tested as possible NIR-triggered photothermal agents in PTT and applied to tumoral treatment and microbial disinfection. Over the past few years, the unique characteristics of gold nanoparticles (AuNPs), due to their pronounced localized surface plasmon resonance, have considerably extended their application in PTT. These nanomaterials can convert the absorbed NIR light into thermal energy with high efficiency, causing local tumor ablation.^9,11,12^ Moreover, PTT can be used to assist common chemotherapy protocols because local hyperthermia can increase vascular and cell membrane permeability, amplifying intratumoral and intracellular chemotherapy drug concentrations.^13,14^

Generally, AuNPs are synthesized through physicochemical methods that require expensive equipment and the use of toxic substances, which restricts their biomedical application.^15^ Since biocompatibility is essential for their application in medical fields, to reduce the toxicity of AuNPs obtained using conventional methodology, functionalization with biocompatible polymers, such as poly(ethylene) glycol,^16^ poly(lactic-*co*-glycolic acid), or hyaluronic acid,^17^ is often required. Recently, researchers have focused on the production of green AuNPs by using eco-friendly and sustainable approaches, replacing common toxic chemicals with substances naturally present in plants, bacteria, and fungi.^18^ Indeed, sugars, tannins, polyphenols, and anthocyanins, generally present in nature, can promote the formation of nanoparticles through very simple thermal syntheses.^15,19^ Agricultural and food waste represent an important source of these compounds, and their recovery and valorization can lead to high added value, reducing environmental impact and disposal costs. Moreover, the conversion of industrial, agricultural or municipal waste into secondary raw materials represents a key aspect of the circular economy focused on recycling, reducing waste, and minimizing environmental impact to create a sustainable future.^20,21^ Different studies have demonstrated the high stability of green AuNPs and good results in PTT applications. In a recent work, *Vaccinium corymbosum* L. extract was used as a natural reducing agent for the synthesis of gold-coated silver nanoparticles (Ag@AuNPs). An evaluation of their biocompatibility and photothermal ability demonstrated a selective dose-dependent cytotoxicity, targeting tumor cells and saving healthy cells such as fibroblasts. Moreover, a heat generation of 44.3 °C was observed after irradiation with a laser at 680 nm for 10 minutes.^22^ Dheyab and co-workers prepared green ultrasmall AuNPs by a sonochemical process using mangosteen peel extracts as starting materials. Viability studies on normal kidney cells and breast cancer cells demonstrated that their cell viability decreases significantly after 808 nm laser irradiation.^23^

In literature, emphasis is placed on green AuNP-mediated PTT in cancer care, while their application in microbial disinfection is less extensively investigated. However, the development of new nanomaterials based on photothermal action, applied to microbial disinfection, represents an interesting extension to the PTT field. Compared to antibiotic treatments generally used in microbial disinfection, PTT drastically reduces the antibiotic resistance effects and the treatment time.^21,24^ Recently, Xu and co-workers prepared modified gold nanorods with selective affinity for bacterial cells and demonstrated their high photothermal conversion efficiency (60%) under NIR laser irradiation and their ability to kill Gram-negative and Gram-positive bacteria.^25^ In another work, Liao synthesized gold nanoworms and demonstrated a dose-dependent thermal increase after 20 min of laser irradiation (808 nm). Their photothermal capacity was evaluated through incubation with *Escherichia coli* and *Staphylococcus aureus*, demonstrating, after six cycles of irradiation, an antibacterial power of >80% and 90%, respectively.^26^

Starting from recent developments in the adoption of green nanoparticle preparation routes,^15,19,27^ in this work, extracts obtained from Lambrusco winery wastes from the Salento area, kindly provided by a local farm (Cantina Vecchia Torre srl), were used to prepare green AuNPs by a simple and eco-friendly thermal process. Indeed, the high amounts of reducing and stabilizing agents that naturally occur in grape marc make their extracts particularly suitable for AuNPs synthesis. After preparation, AuNPs were carefully purified and then characterized by using UV-visible, FTIR, ELS, XRD, and TEM analysis techniques. The biocompatibility of the nanoparticles was studied on human fibroblasts (FibH) and breast cancer cells (MCF-7). Moreover, their photothermal potential was characterized and tested in the MCF-7 cell line as well as in both Gram-positive and Gram-negative bacteria.

## Experimental

### Materials

Gold(iii) chloride trihydrate (HAuCl_4_·3H_2_O) was purchased from Sigma-Aldrich (Steinheim, Germany). Lambrusco grape marc (GM) waste (2024 harvest) obtained from Salento (South of Italy) was kindly provided by a local company (Cantina Vecchia Torre s.*c.a.*, Leverano, Italy). Whatman 1 filters were supplied from Merck KGaA (Darmstadt, Germany). A NEYA 16 R High Speed refrigerated centrifuge (Giorgio Bormac s. r. l, Carpi, Italy) and a PK121 multispeed centrifuge (Thermo Electron Corporation, Waltham, MA, USA) were used for the centrifugation steps. Ultrapure water was obtained using a water purification system (Human Corporation, Seoul, Republic of Korea). Dulbecco’s Modified Eagle Medium/Nutrient Mixture F-12 (DMEM-F12), phosphate-buffered saline solution, ethanol, trypsin, and resazurin were purchased from Sigma-Aldrich (Sintra, Portugal). Fetal bovine serum (FBS) was obtained from Biochrom AG (Berlin, Germany). Cell culture plates, T-Flasks, and other culture plastics were obtained from ThermoFisher Scientific (Porto, Portugal). FibH cells were acquired from PromoCell (Labclinics, S. A., Barcelona, Spain). The MCF-7 cell line was obtained from ATCC (Middlesex, UK).

### Grape marc extract preparation

GM extract was prepared by following the procedure reported in a previous work.^19^ Briefly, 5 g of grape marc powder, obtained after sun drying, was subjected to hydrothermal extraction in 100 mL of ultrapure water at 65 °C for 1 h. After filtration and centrifugation, the extract was stored at −20 °C until use.

### Synthesis and purification of green gold nanoparticles

Green gold nanoparticles (AuNPs@GM) were prepared by following the procedure reported in a previous work with slight variations.^19^ Briefly, an aliquot of GM extract (5 mL) was mixed with an aqueous solution of HAuCl_4_·3H_2_O and heated at 80 °C for 1 h. Nanoparticle formation was confirmed from the color change of the solution (from pale yellow to purple). The crude reaction mixture was purified through several centrifugations at 4000 rpm for 10 minutes to remove larger nanoparticles. Then, all supernatants were collected and centrifuged at 8000 rpm for 10 minutes to remove unreacted products. The obtained pellet was dispersed in water, lyophilized, and stored at 22 °C until use.

### Characterization studies

Purified AuNPs@GM were characterized with a Jasco V-660 UV-visible spectrophotometer (Jasco, Palo Alto, CA, USA). The interaction between the AuNPs and the functional groups of the organic compounds present in the GM extract was evaluated by FTIR analysis with a JASCO 660 plus infrared spectrometer (Jasco, Palo Alto, CA, USA). Electrophoretic light scattering (ELS) was performed with a Malvern Zetasizer Nano ZS 90 (Worcestershire, UK) on diluted samples, in triplicate, to evaluate the zeta potential of the hydrate nanoparticles.

X-ray diffraction (XRD) patterns were collected with a Rigaku MiniFlex diffractometer using Cu Kα radiation (*λ* = 1.5418 Å). The instrument was operated at 30 kV and 15 mA. Data were acquired in the 2*θ* range of 20°–80°, with a step size of 0.02° and a scan rate of 2° min^−1^. These parameters were selected to ensure adequate resolution of the diffraction peaks while maintaining a reasonable acquisition time.

Transmission electron microscopy (TEM) analyses were carried out with a Hitachi H-7700 microscope operating at an accelerating voltage of 120 kV. The gold nanoparticles were deposited onto carbon-coated copper grids by drop-casting from dilute colloidal suspensions and subsequently dried under ambient conditions. To reduce the possibility of electron-beam-induced morphological changes or sintering of the nanoparticles, observations were performed at low electron-beam current densities, and exposure times were kept to a minimum. High-resolution TEM (HRTEM) images were acquired from representative regions to evaluate nanoparticle size and shape, while selected area electron diffraction (SAED) patterns were also recorded to confirm the crystallinity.

### AuNPs@GM photothermal efficiency

AuNPs@GM aqueous solution (400 µg mL^−1^) was prepared and exposed to 10 min of irradiation using an 808 nm NIR laser (1.7 W cm^−2^). Moreover, a thermocouple sensor (accuracy of 0.10 °C) was used to monitor temperature variation.^28^ The AuNPs@GM photothermal conversion efficiency was evaluated using the following equations:

1 *η* = [*h* × *S* × (*T*_max_ − *T*_amb_) − *Q*_dis_]/*I* × (1 − 10^−A808^),

2 *hS* = (*m* × *C*)/*τ*_s_,

where *T*_max_ refers to the peak temperature reached during laser irradiation, while *T*_amb_ corresponds to the room temperature. *Q*_dis_ accounts for the heat dissipated due to absorption by the surrounding medium and container. I represent the NIR laser power density (1.7 W cm^−2^), and A808 corresponds to the optical absorbance of the AuNPs@GM nanostructures at 808 nm. The term *hS* was obtained from eqn (2), where *S* is the surface area of the container, and *h* is the heat transfer coefficient. *C* is the specific heat capacity of water (4.2 J g^−1^ °C^−1^), *m* is the mass of water used (0.2 g), and *τ*_s_ is the time constant of the thermal system, calculated as:

3 *τ*_s_ = −*t*/ln (*θ*),

4 *θ* = (*T* − *T*_amb_)/(*T*_max_ − *T*_amb_),

where *t* is the irradiation duration (600 seconds) and *θ* is a dimensionless temperature parameter derived from eqn (4). The ambient temperature was set at ∼20 °C throughout the experiments.

### Cytocompatibility assay

AuNPs@GM cytocompatibility was evaluated in FibH and MCF-7 cells through a resazurin-based assay.^29^ Briefly, 10 000 cells were seeded per well, in 96-well plates, and cultured with DMEM-F12 medium in a humidified atmosphere (37 °C, 5% CO_2_). After 24 h, the medium was replaced with AuNPs@GM at different concentrations (10–400 µg mL^−1^). Then, 110 µL of resazurin was added to each well at 24, 48, and 72 h of incubation, and cell viability was evaluated by fluorescence measurements of the resultant resorufin using a microwell plate reader (Spectramax Gemini XS, Molecular Devices, LCC) at excitation and emission wavelengths of 560 nm and 590 nm, respectively. In the experiment, positive (K+) and negative (K−) controls were obtained by incubating cells with ethanol (99.9%) and only with culture media, respectively.

### Hemolysis assay

Hemolysis experiments were performed by adapting a method previously described in the literature.^12^ Briefly, fresh EDTA-stabilized blood samples, obtained from adult mice, were centrifuged at 500 rpm for 5 min, at 4 °C, and washed three times with NaCl solution (150 mM) to recover the red blood cells (RBCs). Then, the RBCs were diluted in PBS, distributed in test tubes, and centrifuged. Subsequently, the supernatant was replaced with 1 mL of AuNPs@GM at concentrations of 100, 200, 300, or 400 µg mL^−1^. Moreover, positive (K+) and negative (K−) controls were incubated with Triton-X 100 and PBS, respectively. After 2 and 4 h of incubation, each sample was centrifuged at 500 rpm for 5 min, at 4 °C. Then, 100 µL of supernatants was inoculated into a 96-well plate to measure the adsorption of hemoglobin at 570 nm. Eqn (5) was used to calculate RBC hemolysis percentage:

5 Hemolysis (%) = (*A*_sample_ − *A*_0_)/(*A*_100_ − *A*_0_),

where *A*_100_ and *A*_0_ were the absorbances of the solution at 100% and 0% hemolysis, respectively.

### AuNPs@GM photothermal studies on breast cancer cells

The impact of the AuNPs@GM photothermal capacity on MCF-7 breast cancer cells was evaluated using the resazurin assay.^12^ Briefly, 10 000 cells were seeded in 96-well flat-bottom culture using DMEM-F12. At 24 h, the medium was replaced with AuNPs@GM at a concentration of 10 to 400 µg mL^−1^ and incubated for 24 h. Then, all samples were irradiated for 10 minutes using a NIR laser (808 nm, 1.7 W cm^−2^). The cell viability was determined using the resazurin assay previously described. A positive (K+) and negative (K−) control was obtained by incubating cells with ethanol (99.9%) and only with cell culture, respectively. Moreover, a NIR control (K-NIR) was also considered using irradiated cells without exposure to nanoparticles.

### Antimicrobial properties of AuNPs@GM

AuNPs@GM antibacterial capacity was evaluated using *Staphylococcus aureus* ATCC 25923 and *Escherichia coli* ATCC 25922. Bacteria at a concentration of 5 × 105 CFU mL^−1^ were incubated with AuNPs@GM (100 to 400 µg mL^−1^). After 18 h of incubation with AuNPs@GM, the bacteria were irradiated with the NIR laser (808 nm, 1.7 W cm^−2^) for 10 min. The impact of the AuNPs@GM photothermal effect on the bacterial growth was determined at 24 h of incubation by measuring the OD600. Bacteria without exposure to AuNPs@GM but subjected to NIR laser irradiation were used as the NIR control (K-NIR), while non-irradiated bacteria were designed as the negative (K−) control.

## Results and discussion

### AuNPs@GM preparation and characterization

Plant-mediated synthesis represents a sustainable, low-cost, and eco-friendly alternative to the conventional preparation of AuNPs. GM waste, obtained from Lambrusco winery production, contains high amounts of reducing and stabilizing agents, such as polyphenols, flavonoids, sugars, and anthocyanins, that were easily extracted through a thermal process and used as starting materials for AuNPs@GM preparation.

**Scheme 1:**
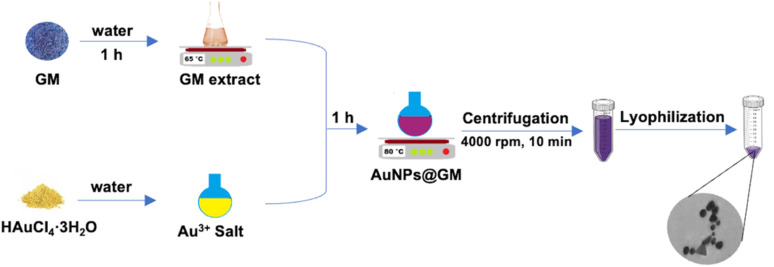
Schematic of the preparation of GM and the synthesis and purification of AuNPs@GM.

In our previous work, grape marc-AuNPs were prepared, and the crude material obtained from the reaction was successfully tested in photocatalytic experiments.^19^ Starting from the growing interest in the development of medical formulations based on gold nanoparticles, in this work, the photothermal conversion ability of AuNPs@GM, as well as its potential antibacterial and antitumoral effects, were investigated. After preparation, AuNPs@GM was purified through different centrifugation steps and washing with water to remove unreacted products and bigger nanoparticles. Such a processing approach led to the selection of gold nanoparticles with a similar size, making them more suitable for biomedical applications (Scheme 1).

**Fig. 1:**
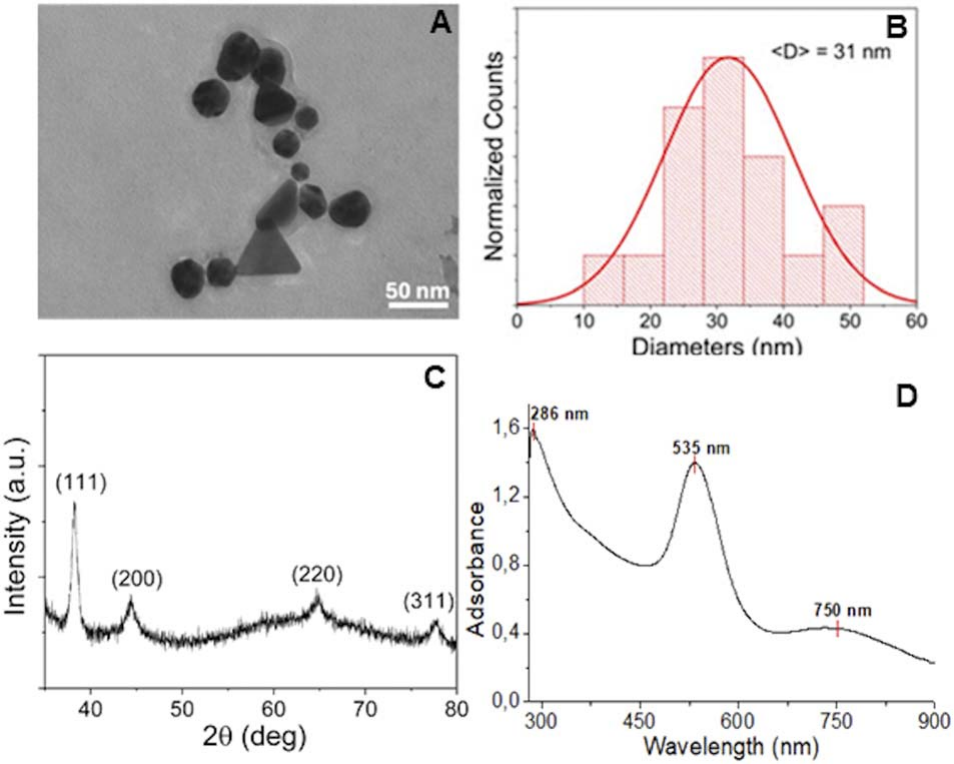
TEM micrograph (A), size distribution histogram (B), XRD pattern (C), and UV-vis spectrum (D) of AuNPs@GM.

TEM analysis was performed to investigate the morphology, dispersion, and size distribution of AuNPs@GM deposited by drop casting onto carbon-coated copper grids. The micrographs (Fig. 1A) revealed particles with spherical or quasi-spherical morphology with uniform contrast, fully consistent with metallic Au. Despite the majority being spherical particles, anisotropic nanoparticles, including triangular and elongated faceted geometries, were also observed in the TEM images. The coexistence of isotropic and anisotropic structures is generally attributed to kinetic factors during nucleation and growth, where local variations in reduction rate, precursor concentration, or surface energy may promote the stabilization of non-spherical morphologies. This may also be related to a preferential interaction and surface accumulation of the GM extract components in specific facets of the gold nanocrystal, guiding the formation of anisotropic shapes. The nanoparticles are generally well-dispersed across the support film, although some localized clustering and short chain-like assemblies are visible. These aggregates likely resulted from solvent evaporation during the drop-casting process rather than intrinsic interparticle interactions. Such observations highlight the importance of sample preparation in influencing apparent dispersion and should be considered when interpreting TEM data. Quantitative size analysis, performed on representative TEM micrographs, yielded the histogram shown in Fig. 1B. The distribution follows an approximately Gaussian profile, centered at an average diameter of ∼30 nm with a modest standard deviation. This indicates a relatively well-controlled synthesis, producing nanoparticles of moderate polydispersity.

**Fig. 2:**
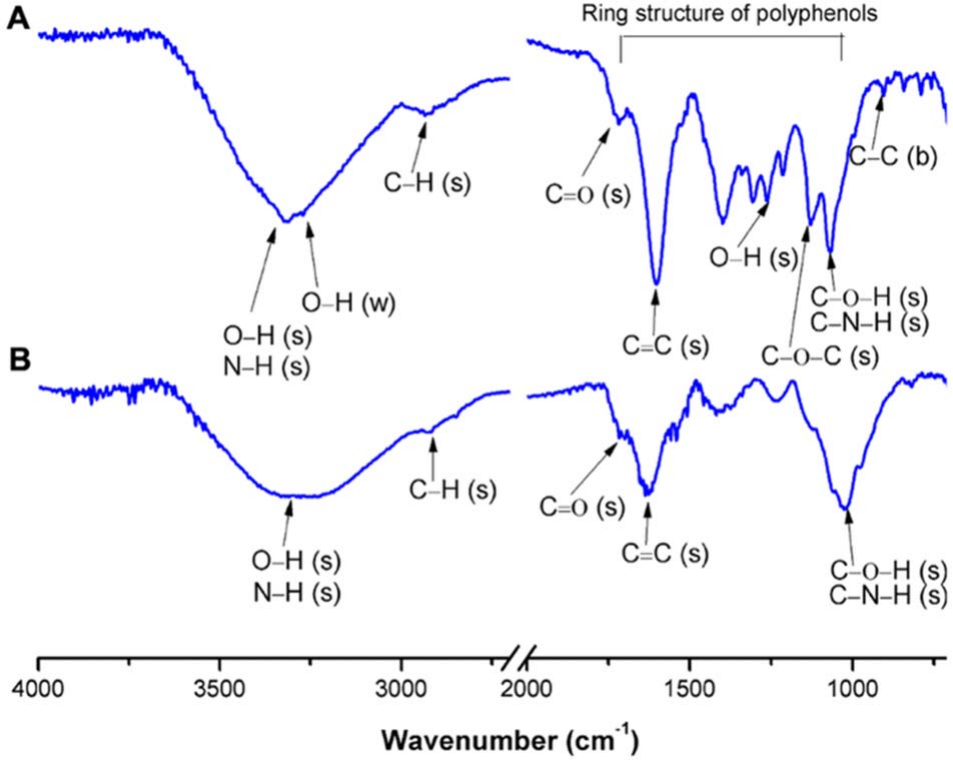
Comparison of the FTIR spectra of the GM extract (A) and AuNPs@GM (B).

Zeta potential analysis, conducted on diluted samples, revealed a negative charge, with a zeta potential of −19.1 ± 2.0 mV, attributed to the presence of hydroxylic groups. Such a value has been associated with prolonged circulation times, with particles showing stability in physiological conditions and reduced opsonization, which is desirable for tumor accumulation *via* the EPR effect.^30^ Furthermore, the XRD findings were in excellent agreement with the TEM analysis. The XRD pattern of AuNPs@GM (Fig. 1C), obtained by drop-casting a colloidal suspension of AuNPs@GM onto a glass microscope slide, clearly confirmed the face-centered cubic (fcc) crystalline structure of metallic gold (space group *Fm*–3*m*). The diffractogram displayed the characteristic set of reflections corresponding to the (111), (200), (220), and (311) planes of fcc Au, with the (111) peak appearing as the most intense, in excellent agreement with the expected relative intensities reported for bulk gold. A pronounced broadening of the diffraction peaks, most notably for the (111) reflection, was evident and fully consistent with nanometric crystallite dimensions. Such line broadening is typically associated with the finite size of coherent diffraction domains, while additional contributions may arise from lattice microstrain. Application of the Scherrer equation, after appropriate correction for instrumental broadening, allowed a reliable estimate of the average crystallite size of about (28 ± 5) nm. Therefore, the convergence of XRD and TEM results provides strong evidence that the sample consists of crystalline, phase-pure gold nanoparticles, predominantly spherical in morphology but containing a minor fraction of triangular and faceted species. AuNPs@GM were also characterized by UV-vis spectroscopy. As shown in Fig. 1D, a plasmonic band at 535 nm confirmed the gold nanoparticles' formation. Moreover, a peak around 286 nm related to the adsorption of phenolic groups of bioactive compounds present in the GM extract can be observed, indicating the presence of a layer of organic matter bound to the surface of the gold nanoparticles. Furthermore, it is also possible to observe a broad absorption band in the NIR-I region, around 750 nm, typically associated with anisotropic gold nanomaterials, such as the triangular and elongated gold nanoparticles observed in the TEM images.^31^ These gold nanoparticles exhibit a longitudinal surface plasmon resonance at longer wavelengths, resulting in increased interaction with light in the NIR region. The appearance of the NIR absorption band is particularly significant because it further supports the applicability of AuNPs@GM as NIR-responsive photothermal agents. The presence of an organic layer on the surface of the gold nanoparticles forms a crucial interface, composed of stabilizers, ligands, and bioactive compounds, that controls the nanoparticle stability, solubility, reactivity, and interactions with their surrounding environment, enhancing their biocompatibility, which is fundamental for biological applications. For this reason, to better clarify the interaction established between bioactive compounds and AuNPs, FTIR analyses were also performed (Fig. 2) in which the spectra of the GM extract (A) and AuNPs@GM (B) were compared. In Fig. 2A, it is possible to observe various broad signals, confirming the complex system of the GM winery extract due to the presence of different bioactive compounds, such as polyphenols, sugars, and tannins. The peak at 3310 cm^−1^ is associated with O–H stretching vibrations, which, coupled with the signal at 1066 cm^−1^ (C–O stretching), indicate the presence of polysaccharides. Furthermore, the same peaks can also be associated with C–N and N–H stretching of aminosugars, as reported by Pelosi and co-workers.^32^ The adsorption peak at 3270 cm^−1^ can be associated with the phenolic O–H wagging vibration, while peaks at 2934 cm^−1^ and 1716 cm^−1^ correspond to the C–H and C=O stretching of olefinic chains and carbonyl groups in esters, respectively.^15,32,33^ Intense bands associated with the ring structure of polyphenols can be observed in the range 1000–1800 cm^−1^. In particular, the peak at 1602 cm^−1^ is assigned to the C=C–O deformation of the heterocyclic C–ring of the flavonoid moiety. The same peak can also be associated with the CH_2_ and CH_3_ of the flavonoid ring.^33^ At 1339 cm^−1^, an adsorption peak related to the C–C stretching of phenols was observed.^32,34^ Moreover, the band at 1252 cm^−1^, attributable to the O–H stretching of phenols, underlines the presence of flavonoid-based tannins in the GM extract, as well as adsorption peaks at 1129 cm^−1^ (C–O weak stretching) and 842 cm^−1^ (C–C–OH vibration), which are also present in the flavanol heterocyclic ring.^35^ Adsorption peaks at 1129 and 1066 cm^−1^ are associated with the C–O–C and C–O–H groups of the phenolic sugars.^33^ Furthermore, the band at 904 cm^−1^ is related to the C–C bending of the heterocyclic ring.^32^

AuNPs@GM showed a spectrum (Fig. 2B) similar to that of the GM extract, with consistent differences in the shape and shift of the signals, especially in the range attributable to polyphenols. The AuNPs@GM spectrum confirms the presence of some of the organic compounds of the extract linked to the purified nanoparticles. A clear change in the shape and a shift of the signals can also be observed by comparing the regions associated with the C–O–C and C–O–H groups of the phenolic sugars. In particular, the disappearance of the peak at 1129 cm^−1^ and a shift of the peak at 1066 cm^−1^ can be observed. Moreover, all regions associated with the polyphenol aromatic structure (1600–1200 cm^−1^) are significantly changed in the AuNPs@GM spectrum. These data confirmed the formation of specific interactions between the polyphenolic compounds present in the extract (*e.g.*, tannins and flavonoids) and AuNPs@GM with a considerable stabilizing effect. The well-known antioxidant properties of these compounds could confer to the gold nanoparticles important anti-inflammatory properties and high biocompatibility that can improve cancer therapeutic approaches.

### Photothermal studies

**Fig. 3:**
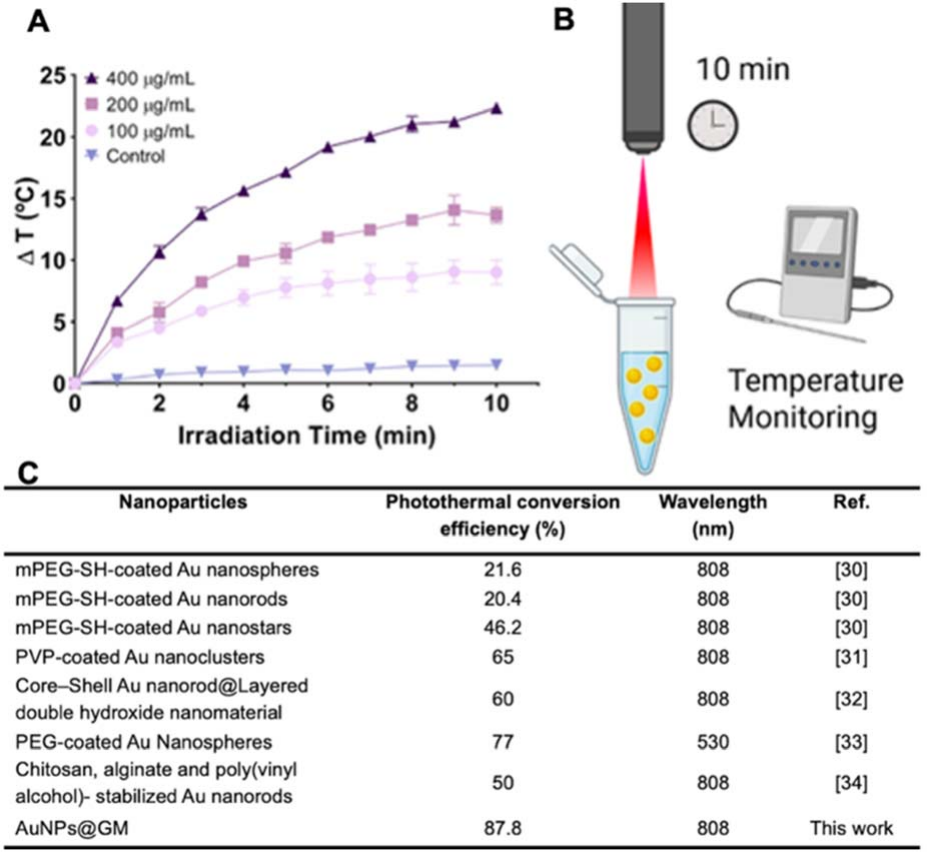
Characterization of the PTT capacity of AuNPs@GM. Temperature variation curves of AuNPs@GM at concentrations of 100, 200, 400 µg mL^−1^ under NIR laser (808 nm, 1.7 W cm^−2^) irradiation for 10 min. The control group is ultrapure water. Data are presented as mean ± s. d. and *n* = 3 (A). Schematic of the PTT process (B). Photothermal conversion efficiency comparison with literature data (C).

The *in vitro* photothermal capacity of AuNPs@GM was evaluated using an 808 nm NIR laser. The results showed that increasing both the concentration and irradiation time increased the heat generation and consequently the final temperature. As can be seen in Fig. 3, irradiation of AuNPs@GM for 10 min results in an increase in the temperature of about 22 °C. Moreover, this capacity remained constant, even with a second and third irradiation, and the final Δ*T* reached 22.1 °C. Further analysis of these results indicated that AuNPs@GM have a photothermal conversion efficiency of about 87.83%. Altogether, these data confirm the photothermal capacity of AuNPs@GM, which is in line with the results and photothermal conversion efficacies of other gold-based nanomaterials.^36–38^ More importantly, the achieved temperature variation indicates that AuNPs@GM, at a concentration of 400 µg mL^−1^, can induce harmful effects on cancer cells, leading to cell membrane disruption, DNA damage, and altered metabolic pathways.^12,39,40^

### Evaluation of cytotoxic effects of AuNPs@GM

**Fig. 4:**
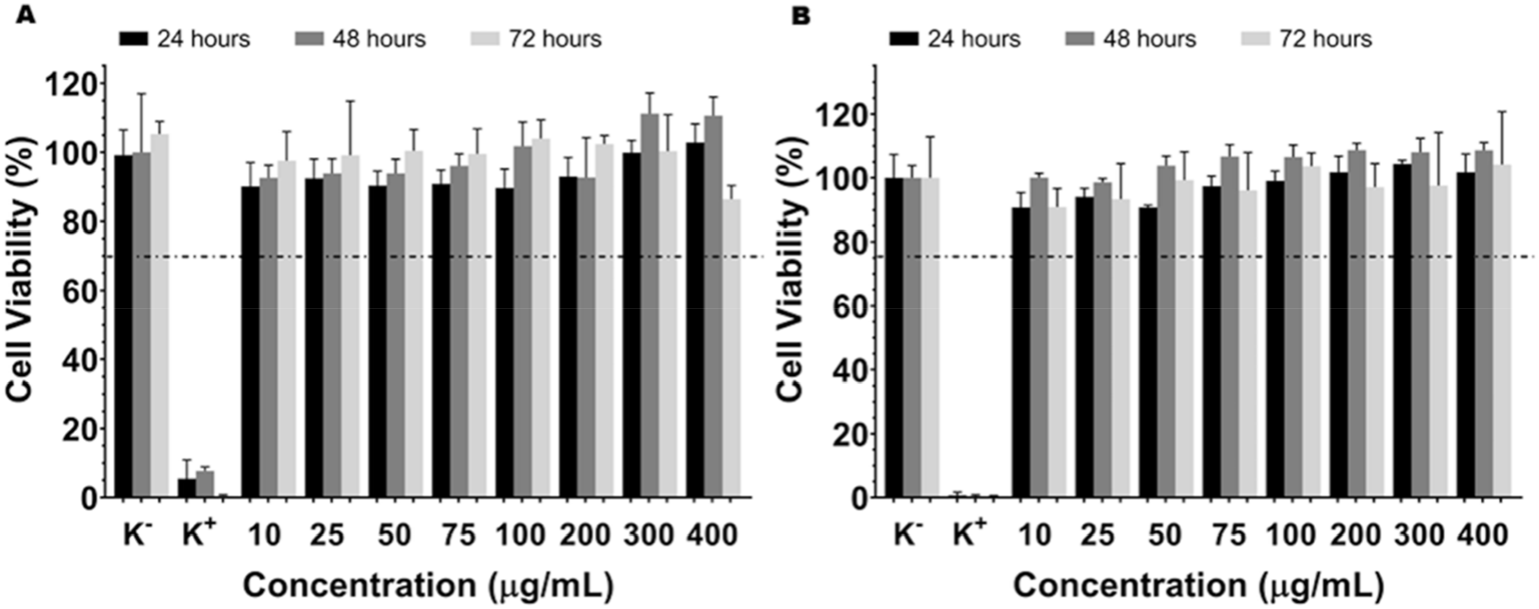
Evaluation of the cytocompatibility of AuNPs@GM at 24, 48, and 72 h in FibH (A) and MCF-7 (B) cells. Positive control (K+): cells treated with EtOH. Negative control (K−): cells without being incubated with nanoparticles. Data are presented as mean ± s. d. and *n* = 5.

The cytotoxicity of AuNPs@GM was evaluated against FibH (Fig. 4A) and MCF-7 (Fig. 4B) cells using the resazurin assay. The results showed that the nanoparticles did not induce cytotoxicity for either of the cell lines. Additionally, according to ISO 10993-5, AuNPs@GM can be considered biocompatible, even at concentrations of 400 µg mL^−1^ since the cell viability remains higher than 70% during the 72 h study. This result can be justified by the presence of the polyphenolic layer around the gold nanoparticles, as previously demonstrated, which form a biocompatible interface, enhancing their biocompatibility compared to common gold nanoparticles prepared using conventional methodologies.

**Fig. 5:**
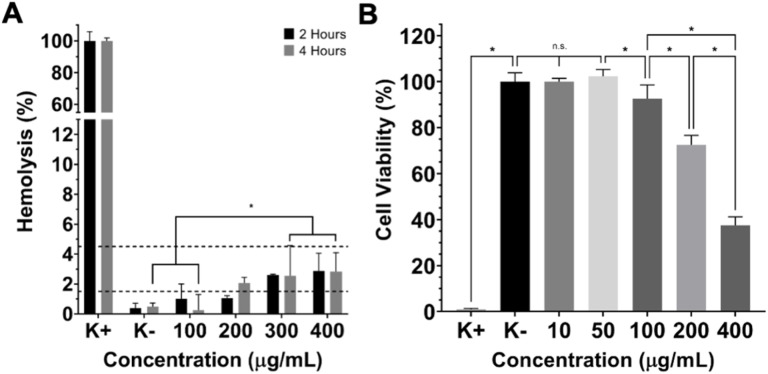
RBC lysis evaluation after incubation with different concentrations of AuNPs@GM (A). Viability of MCF-7 cancer cells after incubation with AuNPs@GM for 48 hours and NIR laser irradiation (10 min, 808 nm, 1.7 W cm^−2^) (B). Data are presented as mean ± s.d., *p < 0.05, and *n* = 5.

To further characterize the biocompatibility of AuNPs@GM, the RBCs' hemolysis was determined at 2 and 4 h, according to ISO/TR 7406, to determine the critically safe hemolytic ratio.^14,41^ As can be observed in Fig. 5A, concentrations above 100 µg mL^−1^ showed a slight hemolytic effect, with 2–3% of the hemoglobin released after 2 and 4 h of incubation.

Hemolysis rates below 5% are typically considered non-hemolytic and biocompatible for most biomedical applications. Therefore, while there is a measurable increase in hemolysis at concentrations above 100 µg mL^−1^, the observed values remain within an acceptable range for *in vitro* blood compatibility. Nevertheless, these moderate hemolytic results can be attributed to increased nanoparticle–membrane interactions at higher concentrations, potentially causing mild disruption of erythrocyte membranes.^42,43^ Nevertheless, these findings still support the overall biocompatibility of AuNPs@GM at relevant concentrations, although further *in vivo* studies and long-term exposure assessments would be beneficial to confirm the safety profile across physiological conditions.

### Therapeutic effect mediated by AuNPs@GM on MCF-7 cells

After the biocompatibility evaluation, the anticancer potential of AuNPs@GM was assessed on MCF-7 cancer cells (Fig. 5B), incubated for 48 h with AuNPs@GM, with or without NIR laser irradiation for 10 min. In Fig. 5B, it can be seen that AuNPs@GM concentrations above 100 µg mL^−1^ lead to a decrease in the viability of the MCF-7 cells. This antitumoral effect is concentration dependent, with AuNPs@GM at 400 µg mL^−1^ reducing the viability of MCF-7 cancer cells to 37%. The results obtained are in agreement with the higher photothermal capacity observed with increasing AuNPs@GM concentrations. In fact, temperatures above 42 °C can induce irreversible cellular damage, making nanoparticle-mediated photothermal therapy an effective approach for targeted cancer treatment by triggering localized hyperthermia and tumor cell ablation. In the future, combinations with other treatment modalities or surface functionalization with targeting moieties may be explored to enhance the therapeutic efficacy of AuNPs@GM and overcome the limitations of monotherapies, addressing challenges such as tumor heterogeneity, drug resistance, and metastasis.^29,40^

### Antibacterial efficacy of AuNPs@GM

**Fig. 6:**
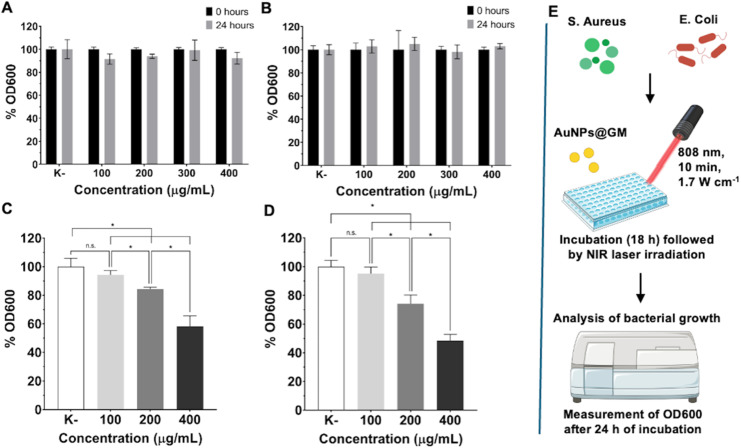
Analysis of the antibacterial capacity of AuNPs@GM against *S. aureus* (A and C) or *E. coli* (B and D) bacteria, without (A and B) and with (C and D) NIR laser irradiation (10 min, 808 nm, 1.7 W cm^−2^). Schematic of the antibacterial assay procedure with NIR laser irradiation (E). Data was normalized with the negative control (K−), bacteria cultured with LB media. Data are presented as mean ± s.d., *p < 0.05, and *n* = 5.

The innate antibacterial capacity of AuNPs@GM was preliminarily screened against *S. Aureus* and *E. Coli* by measuring the OD600. Without NIR laser irradiation, the data show no significant differences in bacterial growth between the control and AuNPs@GM-treated groups (Fig. 6A and B). Therefore, these results suggest that AuNPs@GM alone does not impact bacterial viability or growth.

These results are in agreement with other data in the literature, where gold nanoparticles without additional functionalization or drug loading did not show any significant antibacterial activity.^44,45^ Nevertheless, when irradiated with the NIR laser, AuNPs@GM showed a concentration-dependent antibacterial effect, reaching 41.7% and 52.5% inhibition for *S. aureus* and *E. coli*, respectively, at higher concentrations (Fig. 6C and D). Moreover, the data also revealed that *S. aureus* was slightly more resistant to the PTT treatment. This observation is consistent with previous reports indicating that Gram-positive bacteria, due to their thicker peptidoglycan cell wall, have a higher resistance to thermal damage and to the nanoparticles' penetration, which limits the effectiveness of AuNPs@GM.^45^

Overall, this combination of high-dose biocompatibility, efficient photothermal conversion, and sustainability through waste-derived synthesis differentiates the present material from previously reported seed- and peel-extract-based syntheses of AuNPs, as can be observed in Table 1. In the work of Chen and colleagues, the produced resveratrol-gold nanoparticles at a concentration of 52.78 µg mL^−1^ induced a decrease in the cell viability of both pancreatic normal cells (MS1) and pancreatic cancer cells (BxPC-3) to 72.69% and 67.06%, respectively.^46^ Moreover, Kumar and co-workers described that incubation of grape-generated AuNPs with human breast cancer cells (HBL-100) led to a decrease in cell viability, presenting values close to 80% at the maximum tested concentration of 150 µg mL^−1^.^47^ Chatterjee *et al.* reported in their works the production of AuNPs using *Vitis vinifera* seed extract and *Vitis vinifera* peel extract.^48,49^ The resulting nanoparticles presented a predominantly spherical morphology with sizes ranging from 20 to 55 nm. The *in vitro* studies showed that the AuNPs produced using the peel extract presented an IC50 value of 23.6 µM towards human epidermal carcinoma cells (A431).^49^ In turn, the AuNPs produced with the seed extract did not impact the growth of normal human epidermal keratinocytes (HaCaT), whereas a reduction of the A431 cell viability to ∼50% was observed at a concentration of 25 µg mL^−1^.^48^

**Table 1:** Analysis of previously reported seed- and peel-extract-mediated synthesis of AuNPs

Formulation	Green starting materials	Results	Ref.
Resveratrol-AuNPs	Grape skin extract	Size: 11.9 nm. Zeta potential: 66.7 mV. Dosage of 52.78 µg mL^−1^ decreases cell viability of MS1 and BxPC-3 cells to 72.69% and 67.06%, respectively	46
Grape-AuNPs	Grape extract, glutathione and lipoic acid	Size of grape-AuNPs: 20–45 nm. Size of modified grape-AuNPs with glutathione and lipoic acid: 40–80 nm. Dosage of 150 µg mL^−1^ decreases cell viability of HBL-100 cells to close to 80%	47
*Vitis vinifera*-AuNPs	*Vitis vinifera* seed extract	Size: 40–55 nm. Zeta potential: −15 mV. Dosage of 50 µM does not impact the growth of HaCaT cells. Dosage of 25 µg mL^−1^ decreases cell viability of A431 cells to ∼50%	48
*Vitis vinifera*-AuNPs	*Vitis vinifera* peel extract	Size: 20–40 nm. Dosage of 23.6 µM decreases cell viability of A431 cells to 50%	49
*Vitis vinifera*-AuNPs	*Vitis vinifera* peel and seed extract	Size: 40–60 nm. Dosage of 1, 2, 3, and 4 mg/kg/b wt./animal/day for 30 days does not induce significant toxicity in swiss albino mice. *Vitis vinifera*-AuNPs suppressed abnormal skin cell proliferation that occurred during induced skin papillomagenesis, revealing a chemopreventive capacity	50
Grape pomace-AuNPs	Grape pomace wastewater extract	Size: 30 nm. Grape pomace-AuNPs attenuated H_2_O_2_-induced growth inhibition and exhibited scavenging activity against the intracellular ROS, supporting the normal growth of normal human dermal fibroblasts	51
AuNPs@GM	Grape marc extract	Size: 30 nm. Zeta potential: −19 mV. RBC hemolysis is below 5%. Dosage of 400 µg mL^−1^ does not induce toxicity in FibH and MCF-7, but it decreases cell viability of MCF-7, *E. coli* and *S. aureus* to 37%, 52.5% and 41.7%, respectively, under NIR laser irradiation	This work

## Conclusions

In this work, winery waste valorisation was achieved through the development of a sustainable approach to prepare new gold-based biocomposite nanomaterials with interesting results in the PTT field. The synthesized AuNPs@GM were composed of spherical, triangular, and elongated rod-like nanoparticles with a size distribution of ∼30 nm. Physicochemical characterization further revealed the presence of a superficial layer of polyphenolic compounds, such as tannins and flavonoids, from the GM extract on the surface of the nanoparticles that enhance their biocompatibility. The *in vitro* studies further confirmed the cytocompatibility of AuNPs@GM, despite registering hemolysis values of ∼3% at the highest concentration. Additionally, AuNPs@GM also showed potential for the NIR-triggered PTT of cancer and bacterial infection, impacting cellular growth and mediating cellular death. Altogether, our data shows the potential biomedical application of AuNPs@GM. The study also contributes to the development of more environmentally friendly production methods for gold nanoparticles, in line with the circular economy principles, through the valorisation of GM agricultural waste and environmental impact reduction. Future work will focus on the relationship between GM extract composition and gold nanoparticle shape, aiming to identify the main components responsible for gold nucleation or even guiding particle growth. These studies will contribute towards the development of more controlled synthesis procedures and present alternatives that could direct the growth of nanoparticles to different morphologies.

## Author contributions

Lucia Mergola: conceptualization, data curation, formal analysis, investigation, methodology, project administration, supervision, visualization, writing-original draft, and writing-review and editing. André F. Moreira: conceptualization, data curation, formal analysis, investigation, and writing-review and editing. Sónia P. Miguel: formal analysis, investigation, and writing-review and editing. Paula Coutinho: conceptualization, resources, and writing-review and editing. Christian Demitri: investigation, visualization, and writing–review and editing. Antonio Serra; data curation, formal analysis, and writing-review and editing. Daniela Manno: data curation, formal analysis, and writing-review and editing. Alessandro Buccolieri: data curation, formal analysis, and writing-review and editing. Roberta Del Sole: conceptualization, data curation, formal analysis, funding acquisition, project administration, resources, supervision, writing-original draft, and writing–review and editing.

## Conflicts of interest

There are no conflicts to declare.

## Data Availability

The data supporting the findings of this study are available from the corresponding authors upon reasonable request.
